# Supplementary material for: The impact of school closure intervention during the third wave of the COVID-19 pandemic in Italy: Evidence from the Milan area
Source: PLoS One. 2022 Jul 12;17(7):e0271404. doi: 10.1371/journal.pone.0271404 (PMC9275695; doi:10.1371/journal.pone.0271404)
Supplement: S1 File — (DOCX) [file pone.0271404.s001.docx]

**Supplementary Material**

**Table S1: Distribution of COVID-19 cases, swab tests performed, and positivity rate before and after the school closure intervention (Pre/Post March 5).**

|  | **COVID-19 cases (n)** | | | **Swab tests (n)** | | | **Positivity rate (%)** | | | **Population**  **(n)** |
| --- | --- | --- | --- | --- | --- | --- | --- | --- | --- | --- |
|  | ***Total*** | ***Pre*** | ***Post*** | ***Total*** | ***Pre*** | ***Post*** | ***Total*** | ***Pre*** | ***Post*** |  |
| *3–11 y.o.* | 2,859 | 1,485 | 1,374 | 44,174 | 26,095 | 18,079 | 6·5 | 5·7 | 7·6 | 281,126 |
| *12–19 y.o.* | 4,733 | 2,204 | 2,529 | 50,950 | 24,812 | 26,138 | 9·3 | 8·9 | 9·7 | 257,171 |
| *20+ y.o.* | 49,739 | 19,881 | 29,858 | 681,410 | 305,384 | 376,026 | 7·3 | 6·5 | 7·9 | 2,837,675 |
| *Total* | 57,331 | 23,570 | 33,761 | 776,534 | 356,291 | 420,243 | 7·4 | 6·6 | 8·0 | 3,375,972 |

**Figure S1: Plot of residuals against time with locally weighted regression curve, ATS (without Bollate), individuals aged 3–11 years old (cut-off: six days).**

*
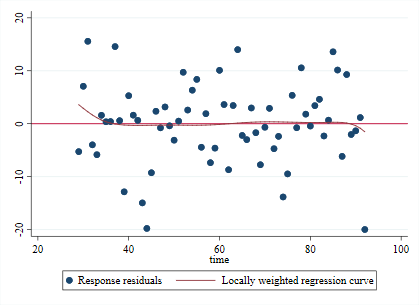
*

**Figure S2: Plot of residuals against time with locally weighted regression curve, ATS (without Bollate), individuals aged 12–19 years old (cut-off: six days).**

*
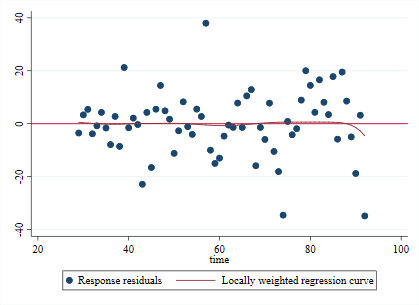
*

**Figure S3: Plot of residuals against time with locally weighted regression curve, ATS (without Bollate), individuals aged 20+ years old (cut-off: six days)***.*

*
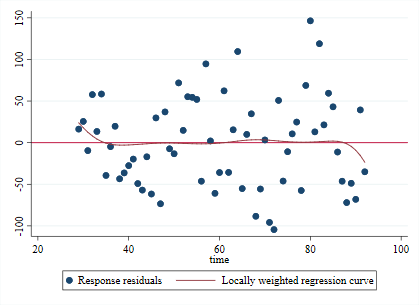
*

**Figure S4: Autocorrelation of residuals, ATS (without Bollate), individuals aged 3–11 years old (cut-off: six days).**

*
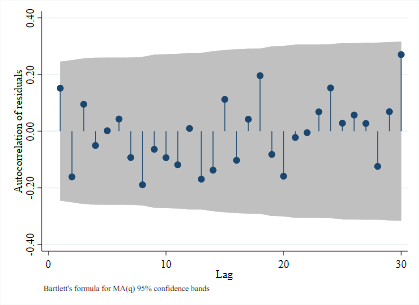
*

**Figure S5: Autocorrelation of residuals, ATS (without Bollate), individuals aged 12–19 years old (cut-off: six days).**

*
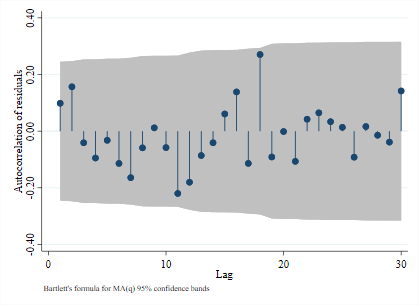
*

**Figure S6: Autocorrelation of residuals, ATS (without Bollate), individuals aged 20+ years old (cut-off: six days).**

*
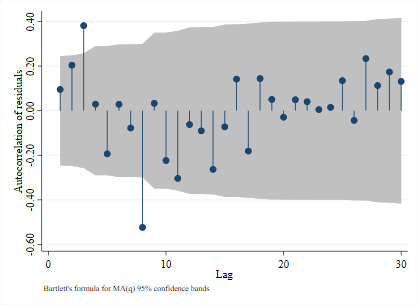
*

**Figure S7: Partial autocorrelation of residuals, ATS (without Bollate), individuals aged 3–11 years old (cut-off: six days).**

*
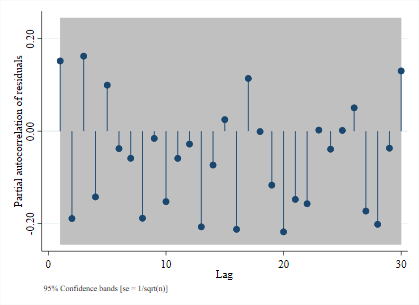
*

**Figure S8: Partial autocorrelation of residuals, ATS (without Bollate), individuals aged 12–19 years old (cut-off: six days).**

*
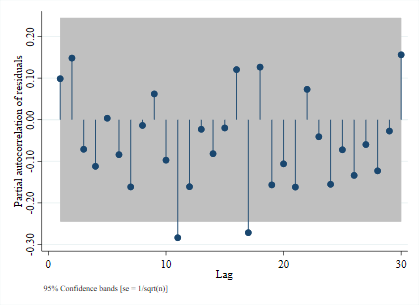
*

**Figure S9: Partial autocorrelation of residuals, ATS (without Bollate), individuals aged 20+ years old (cut-off: six days).**

*
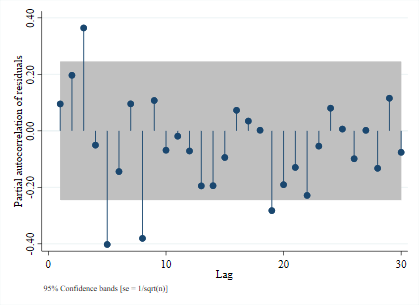
*

**Figure S10: Seasonality-adjusted model with Fourier terms, ATS (without Bollate), individuals aged 3–11 years old (cut-off: six days).**

*
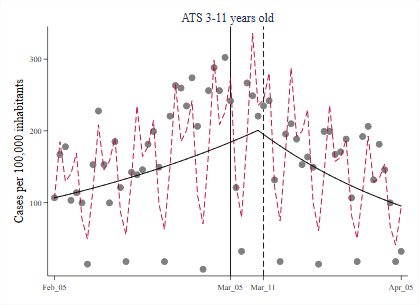
*

**Figure S11: Seasonality-adjusted model with Fourier terms, ATS (without Bollate), individuals aged 12–19 years old (cut-off: six days).**

*
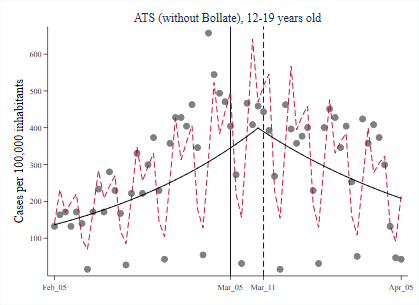
*

**Figure S12: Seasonality-adjusted model with Fourier terms, ATS (without Bollate), individuals aged 20+ years old (cut-off: six days).**

*
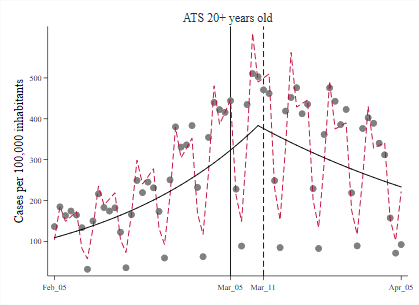
*

**Table S2: Poisson regressions of daily incident COVID-19 cases, segmented with a 14-day cut-off after the school closure intervention. ATS (without Bollate).**

|  | **3–11 y.o.** | | | **12–19 y.o.** | | | **20+ y.o.** | | |
| --- | --- | --- | --- | --- | --- | --- | --- | --- | --- |
|  | **IRR** | **P-value** | **[95% C.I.]** | **IRR** | **P-value** | **[95% C.I.]** | **IRR** | **P-value** | **[95% C.I.]** |
| **Cut-off*time** | 0·96 | 0·012 | [0·94–0·99] | 0·96 | 0·017 | [0·93–0·99] | 0·97 | 0·000 | [0·96–0·98] |
| **Time** | 1·02 | 0·001 | [1·01–1·04] | 1·03 | 0·000 | [1·01–1·05] | 1·02 | 0·000 | [1·01–1·03] |
|  |  |  |  |  |  |  |  |  |  |
| **Scenario** |  |  |  |  |  |  |  |  |  |
| *1 - 'yellow' (ref.)* | – | 1 | – | – | 1 | – | – | 1 | – |
| *2 - 'orange'* | 0·97 | 0·802 | [0·74–1·27] | 0·95 | 0·726 | [0·74–1·24] | 0·91 | 0·166 | [0·81–1·04] |
| *3 - 'strengthen orange'* | 0·71 | 0·046 | [0·51–0·99] | 0·68 | 0·018 | [0·50–0·94] | 0·81 | 0·004 | [0·71–0·94] |
| *4 - 'red'* | 0·52 | 0·001 | [0·35–0·76] | 0·56 | 0·002 | [0·38–0·81] | 0·67 | 0·000 | [0·57–0·79] |
|  |  |  |  |  |  |  |  |  |  |
| **Weekday** |  |  |  |  |  |  |  |  |  |
| *Sunday (ref.)* | – | 1 | – | – | 1 | – | – | 1 | – |
| *Monday* | 10·53 | 0·000 | [5·89–18·82] | 6·33 | 0·000 | [3·58–11·21] | 1·43 | 0·040 | [1·02–2·00] |
| *Tuesday* | 10·12 | 0·000 | [5·98–17·11] | 5·51 | 0·000 | [3·26–9·30] | 2·12 | 0·000 | [1·62–2·76] |
| *Wednesday* | 8·67 | 0·000 | [5·34–14·08] | 5·34 | 0·000 | [3·26–8·74] | 2·04 | 0·000 | [1·59–2·60] |
| *Thursday* | 8·13 | 0·000 | [4·89–13·51] | 4·91 | 0·000 | [2·90–8·31] | 1·85 | 0·000 | [1·44–2·37] |
| *Friday* | 8·86 | 0·000 | [5·30–14·79] | 5·19 | 0·000 | [3·06–8·83] | 1·80 | 0·000 | [1·39–2·33] |
| *Saturday* | 6·20 | 0·000 | [3·84–10·00] | 3·95 | 0·000 | [2·47–6·33] | 1·89 | 0·000 | [1·60–2·24] |
|  |  |  |  |  |  |  |  |  |  |
| **Swab tests (ATS)** | 1·00 | 0·280 | [1·00–1·00] | 1·00 | 0·001 | [1·00–1·00] | 1·00 | 0·000 | [1·00–1·00] |
| **New cases (Italy)** | 1·00 | 0·348 | [1·00–1·00] | 1·00 | 0·643 | [1·00–1·00] | 1·00 | 0·566 | [1·00–1·00] |
| **Positivity rate (Italy)** | 0·95 | 0·333 | [0·86–1·05] | 0·95 | 0·279 | [0·86–1·05] | 1·05 | 0·075 | [0·99–1·11] |

**Figure S13: Model with a 14-day cut-off, 3–11 years old. Solid line: predicted trend based on the covariate-adjusted segmented Poisson regression model. Dashed line: unadjusted trend.**

*
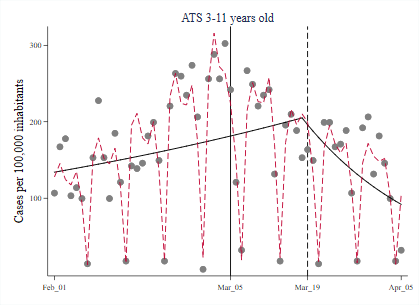
*

**Figure S14: Model with a 14-day cut-off, 12–19 years old. Solid line: predicted trend based on the covariate-adjusted segmented Poisson regression model. Dashed line: unadjusted trend.**

*
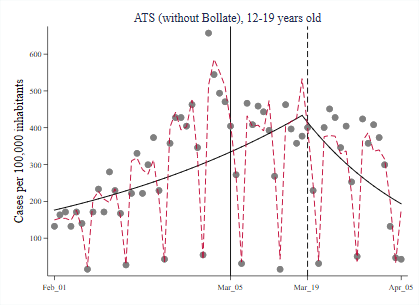
*

**Figure S15: Model with a 14-day cut-off, 20+ years old. Solid line: predicted trend based on the covariate-adjusted segmented Poisson regression model. Dashed line: unadjusted trend.**

*
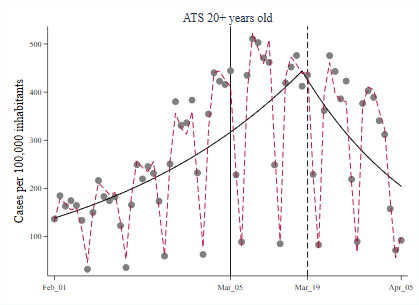
*
