# Supplementary material for: The impact of school closure intervention during the third wave of the COVID-19 pandemic in Italy: Evidence from the Milan area
Source: PLoS One. 2022 Jul 12;17(7):e0271404. doi: 10.1371/journal.pone.0271404 (PMC9275695; doi:10.1371/journal.pone.0271404)
Supplement: Attachment — Submitted filename: Response to Reviewers.docx [file pone.0271404.s003.docx]

Dear Reviewers,
We thank you for your comments and suggestions, which we believed have helped us in reaching a higher quality output. We provide point-by-point answers to each comment below.

Overall, we made efforts to make our findings more comprehensible to the readers, as we believe that in the first version we did not stress enough the importance of the temporal dimension in suggesting the relevance of the school closure intervention. It is not so much the magnitude of the coefficients that convinced us about the effectiveness of the intervention, but rather the fact that the contagion curve started to decrease first in those directly affected by the intervention, and only subsequently in the adult population, which benefited from the reduction in contagion in school-aged children, only marginally affected by the virus before the novel “British” strain.

Despite we included several time-varying confounders, we agree with the reviewers that this might not be enough to completely separate the effect of the intervention from other unmeasured concomitant variations, and then that the decrease in the contagion curve could be attributable to factors other than the school closure. However, the temporal order of contagion decrease in different age groups is a very relevant point in favor of the effectiveness of the intervention, as we found no other explanation for this robust and meaningful evidence.

Hence, we deeply revised to manuscript to highlight this issue.

Yours sincerely,
The authors

**Reviewer #1:** I would like to congratulate the authors for the study as it would help to take action by the concerned authorities in preventing the spread of the COVID-19 and in pandemics in future too. Though there could be many confounding factors and bias in the decreasing hospitalization and casualties that the authors had mentioned in the limitation. There are also other school of thoughts on the risk vs benefit of social costs and other concerns of school closure.

Overall the study is appreciable in terms of analysis, interpretation and publucatiton.

We would like to thank the reviewer for his/her appreciation. We are aware of the potential problems with time-varying confounding and we did our best to try to minimize this source of bias.

We also agree that from a perspective of policy implementation it is not an easy task to weigh costs and benefits of such an intervention, however our analysis was limited to assess the effectiveness of the intervention in a purely epidemiological perspective, focusing on the reduction of contagion, something that in the Italian context was questioned without basing on empirical evidence.

**Reviewer #2:** Report for “The impact of school closure during the third wave of the COVID-19 pandemic in Italy: evidence from the Milan area.”

This study evaluates the impact of school closure policy on reducing the spread of COVID-19 pandemic. The authors use an interrupted time series design by comparing right before and right after the implementation of the policy.

Major comments:

1. As acknowledged by the authors, it is very hard to control for other concurrent policies or events in this design. Is it possible to find a region that is close to the Milan area but didn’t implement the policy on March 5th to serve as a control group? We can show that the infection in the control region didn’t change much after March 5th. By doing so, we can at least control for factors such as national level policy variation, change in climate and people’s awareness.
Unfortunately, we have access to individual data concerning exclusively Milan ATS. The only thing we could do is to compare the overall trend in incident cases (not disaggregated by age), by means of national open data aggregated by region.

From the following figure, regarding the overall incident cases (all ages group) in the whole Country in the same period, it is evident that the trend is different from the one detected in our study area, whereby in correspondence of our school closure cut-off (which in the Milan area was followed by reduction in contagion) in Italy the contagion continued to increase, starting to decrease very slowly only subsequently.

This is even clearer focusing on the Lombardy Region and on those regions located in its surrounding: Except for Emilia-Romagna, which shows a similar trend to the one observed in Lombardy, the other regions show completely different trends, despite their proximate location. Hence, we have reason to believe that the local trend detected was hardly attributable to the overall national epidemic trend. As regards the Emilia-Romagna case, though we have no deep knowledge of it as for the Lombardy case, a quick search revealed that the region entered in “red zone” in March 6 to stop the circulation of the virus “also among the youngest”, hence the similarity with our case (<https://www.regione.emilia-romagna.it/notizie/2021/marzo/covid-citta-metropolitana-di-bologna-e-provincia-di-modena-dal-4-marzo-in-zona-rossa-scuole-chiuse-ma-nidi-e-materne-fermi-dal-6>).

Indeed, given the locally based Italian National Healthcare System, policies were issued at the regional level. As explained in the text, we choose a specific time period in which the only relevant variation in terms of COVID-19 containment measures was attributable to the school closure intervention, explicitly to minimize bias. Climate was fairly stable during the study period and the study area is characterized by the same environment (the Po Valley). There is no data concerning people's awareness, but it is reasonable to expect that this remained stable in such a limited period.

Average = 7.5

Average temperature in Milan February 2021

|  |  |
| --- | --- |
| 2021-02-01 | 6 |
| 2021-02-02 | 4 |
| 2021-02-03 | 6 |
| 2021-02-04 | 8 |
| 2021-02-05 | 8 |
| 2021-02-06 | 9 |
| 2021-02-07 | 8 |
| 2021-02-08 | 8 |
| 2021-02-09 | 9 |
| 2021-02-10 | 8 |
| 2021-02-11 | 7 |
| 2021-02-12 | 3 |
| 2021-02-13 | 0 |
| 2021-02-14 | 0 |
| 2021-02-15 | 2 |
| 2021-02-16 | 5 |
| 2021-02-17 | 8 |
| 2021-02-18 | 8 |
| 2021-02-19 | 9 |
| 2021-02-20 | 10 |
| 2021-02-21 | 10 |
| 2021-02-22 | 10 |
| 2021-02-23 | 10 |
| 2021-02-24 | 10 |
| 2021-02-25 | 11 |
| 2021-02-26 | 11 |
| 2021-02-27 | 12 |
| 2021-02-28 | 9 |

Average = 9.7

Average temperature in Milan March 2021

|  |  |
| --- | --- |
| 2021-03-01 | 8 |
| 2021-03-02 | 8 |
| 2021-03-03 | 9 |
| 2021-03-04 | 9 |
| 2021-03-05 | 12 |
| 2021-03-06 | 8 |
| 2021-03-07 | 6 |
| 2021-03-08 | 7 |
| 2021-03-09 | 8 |
| 2021-03-10 | 8 |
| 2021-03-11 | 8 |
| 2021-03-12 | 11 |
| 2021-03-13 | 9 |
| 2021-03-14 | 10 |
| 2021-03-15 | 10 |
| 2021-03-16 | 12 |
| 2021-03-17 | 10 |
| 2021-03-18 | 7 |
| 2021-03-19 | 6 |
| 2021-03-20 | 6 |
| 2021-03-21 | 5 |
| 2021-03-22 | 8 |
| 2021-03-23 | 9 |
| 2021-03-24 | 10 |
| 2021-03-25 | 11 |
| 2021-03-26 | 13 |
| 2021-03-27 | 13 |
| 2021-03-28 | 13 |
| 2021-03-29 | 15 |
| 2021-03-30 | 16 |
| 2021-03-31 | 16 |

2. Another way to clarify the mechanism is to investigate whether the effect of the policy is more significant in regions with more schools. If the effect is really driven by school closure, I think this should be the case. This is a relevant suggestion, but we believe that such a fine-grained analysis would go beyond the scope of this study. Moreover, the study area is the third in the country for population density, hence we do not expect to find relevant internal differences as concerns the presence of schools in the territory.

3. School closure may force some parents to stay at home and take care of their children, which will also reducing transmission. This can also become a mechanism through which school closure affect transmission. This is a very relevant issue, we already thought about the existence of such a mechanism. However, the fact that the contagion started do decrease first in school-aged children and only subsequently in the adult population should exclude this possibility as a major cause of reduction in the spread of the virus. We added a passage at pages 10-11 to clarify this issue: “The temporal order of the events should also exclude the possibility of other causation mechanisms, such as a decrease in contagion as a consequence of parents forced to stay at home to take care to their children, as otherwise the variations in the age-stratified epidemic curves should be concomitant.”

**Reviewer #3:** Referee report for “The impact of school closure during the third wave of the COVID-19 pandemic in Italy: evidence from the Milan area”

Summary: The authors attempt to address the important question of the effect of school closures on the spread of COVID-19, focusing on the Lombardy area in Italy during an outbreak in Feb – Apr 2021. The authors concentrate on children ages 3 to 11, whose schools were shut down on March 5th until April 5th. Older students’ schools had already been shut down for a couple of weeks by the time the 3-11 year-olds’ schools were closed. The authors estimate Poisson regression models for COVID cases for 3-11 year-olds and 20+ year-olds separately (also 12-19 in an appendix), pursuing an interrupted time-series research design, allowing for a slope break 6 days after the schools closed. They find that after the schools shut down, case counts decreased by about 4% for 3-11 age group and 3% for the 20+ group.

While the authors do not test for differences between estimates in these two groups,

it seems quite clear from their results and plots that these 4% and 3% estimates are not statistically distinguishable. I would argue they represent the same effect. The authors then conclude that, “it is nonetheless clear that school closure contributed substantially to mitigate the spread of the Alpha variant among the population” (p. 15).

This, together with some subsequent comments, helped us in realizing that we have not been sufficiently clear in explaining our results throughout the text. We believe we had some more convincing evidence with some material we initially put only in the Appendix, and we agreed to move to the main text, which has been revised deeply according to this issue.

Our main objective was to demonstrate the effectiveness of the school closure intervention in reducing contagion among the overall population. We stratified our analyses by age groups given that such groups were affected by the intervention with different timing. Hence, we hypothesized (and the data supported our guess) that different age groups would have been touched by the intervention in different times. Our primary aim was not to compare the effectiveness of school closure between age groups. The most important element in support of our findings is the temporal dimension of the reduction in contagion. Looking at the raw distribution of daily cases (Figure 1) and the survival curves model (first in the Appendix, now moved in the main text), it is clear that contagion started to decrease first in the 3-11 population directly affected by the intervention, and only subsequently in the adult population. Hence, breaking contagion in schools, the spread started to decrease also within families. If the intervention was not effective, we would have expected contagion to increase all age groups. If the contagion was instead already decreasing for reasons other than the intervention, then the temporal order of contagion reduction in different age classes remains unexplained.

Comments

1) I think the authors address an important question and they have some nice data on covid cases by age in the Milan, Italy area.

2) That said, I do not think the authors employ a credible research strategy in this case with their interrupted time series. This is an obsolete strategy and should be replaced with more modern approaches. In the authors’ case, a difference-in-differences (DD)/event study approach would be a modern and credible research design that I think could be feasible for the authors given their data. The problem with the interrupted time series approach is the authors can’t account for other factors that could be correlated with time. Other COVID-19 precautions implemented at the same time as the school closures are one of these other factors. The authors discuss these and clam they are likely to have little effect (without evidence). They might have a point, but that doesn’t mean that we shouldn’t use available methods to be more confident, rather than just having to take the authors’ words for it. More important, though, is the fact that populations respond strongly to COVID-19 out-breaks on their own, without the government having to force them to change their behavior. We have seen this in many ways during the COVID-19 pandemic. Thus, while the government responds to the covid outbreak, so do the people in changing their behavior. This change in behavior could be the driving factor for the fall in cases (or a contributor). The authors’ interrupted time series does not allow for any way to control for these factors.

We agree with the reviewer that usually there are some more innovative techniques to assess intervention effectiveness; however, after considering them we had to exclude them for the purpose of this study. Moreover, we are firmly convinced that there are no “outfashioned” techniques as long as they prove to be effective. Leading journals such as the *International Journal of Epidemiology* and *Journal of Clinical Epidemiology* published articles to encourage the use of the interrupted time-series design in recent years, which we followed to adopt a thorough methodology:
<https://doi.org/10.1016/j.jclinepi.2018.05.026>
<https://doi.org/10.1093/ije/dyw098>
<https://doi.org/10.1093/ije/dyy135>

We made efforts to keep under control possible time-varying confounders, choosing a time frame with a stable background of interventions. The only major difference between the pre- and the post-intervention period was indeed the school closure intervention. Nonetheless, we introduced in our model several covariates to minimize confounding. However, not being this an experimental design, the presence of omitted variables bias and unobserved confounding is somehow unavoidable. Like in every statistical model, we had to deal with uncertainty, try to minimize it, and make it explicit in our discussion, as we hope we did. Moreover, DiD is subject to the similar limitations and assumptions to time-series design as regards the presence of time-varying confounding.

We believe that people's behavioral change did not play a role at all. Otherwise, why did the Italian Government have to deal with the “green pass” (Digital Covid Certificate), and to make vaccination compulsory for same working categories and age classes? We personally witnessed parents’ demonstrations against school closure in the Milan ATS headquarter, which are not exactly representative of spontaneous change in behaviors (see: <https://www.repubblica.it/cronaca/2021/03/26/news/scuola_l_italia_in_dad_scende_in_piazza_manifestazioni_in_60_citta_-293919748/>). Structural interventions are specifically conceived to “force” the population to adopt safe behaviors, when it is impossible to contain the outbreak otherwise.

3) The authors would be much better trying to provide a control group for comparison, as this group would control for the behavior of the population, the other government restrictions, and any other omitted factors. Looking at their data, it seems like the authors could have used 12-19 year-olds as such a group, or even the 20+ year-olds, though 12-19 is the better group as most of that group also attends school. The authors note in Table 1 that with entry into the “yellow zone” on February 1, schools at the second level of secondary school and above (i.e., 12 year-olds and up) had their schools closed. So why not compare cases among this group to those of 3-11 year-olds who schools weren’t closed? The 3-11 age group would control for population response to covid and also for the other government restriction that were imposed. The difference in cases between the groups could then more credibly be called the effect of school shut-down. They could even use variation from when the younger kids’ schools were shut down as part of this approach, as well. Alternatively, the authors could use the 20+ year-olds as a control group. This is not as strong a control group since these people don’t attend school, but it is far better than the approach the author currently use where there is no control for population response at all.

In our scenario there is no way to identify controls. In experimental and non-experimental studies, age is the first confounding factor to keep under control in order to enable comparison. We do not think it is feasible to build control groups with individuals sharing completely different susceptibility and risk profiles with respect to the outcomes. We believe it is not logical to compare kids, which generally are less susceptible to contagion (except for the novel variant) and live mostly in the school environment, with adolescents, which are engaged in more social life beyond the school environment, and also with adults who have an heterogenous and completely distinct lifestyle, depending on their daily and working habits. Also, as highlighted above, our primary goal is to evaluate the effect of the intervention on the overall population, not between different age groups. We revised the text in several points to make this clearer.

Moreover, as reported in the text, the 12-19 group was subject to an alternation of school opening and closure, sometimes with blended learning (in person and remote). This is another reason for the impossibility to use it as a control group. Though this may not be the most orthodox method, the only way to approach the issue was to find a stable period as regards background interventions and examining contagion trends before and after it.

Nonetheless, to our knowledge there are no experimental studies on this topic in the current literature, given the natural setting of the event.

4) Looking at the authors’ data, we can informally perform a sort of DD approach by comparing how case rates changed over time across these three groups. The results are presented in Table 2 and in Table S2 (in the appendix). While the authors don’t formally test for differences, it is clear that the estimates of spread in the authors’ pre-period (estimate for variable “time”) are almost quantitatively identical. Moreover, during the post-period, they are still almost quantitatively the same. Without a doubt, across the groups these estimates would not be distinguishable statistically. Thus, in my view the authors’ data is suggestive of their being no effect of the school shut-downs. That said, if the authors had performed a more formal DD style analysis, maybe they would have been able to show a difference. But at this point, I see their analysis as suggesting no effect.

We believe the answer to this point has been already addressed in other answers above. Moreover, the 4% and 3% represent a daily reduction of cases. This implies that, for instance, that at 7, 14, and 21 days from the intervention, the differences outlined are much wider and significant. We computed predicted probabilities from the models to estimate the weekly mean cases net of confounders, which in 3-11 age group were 62 in the week before school closure, and fell to 51, 43, 37 in the weeks following the interventions. In the 20+ age group the mean of daily cases was 928 before the intervention, and then 1096, 976, 898 in the weeks after, indicating a delayed effect with respect to the youngest group. We also plotted fractional polynomial (degree=2) of the predicted probabilities from the models (normalized to vary between 0-1, as the absolute numbers are quite different between age classes). From the graph it is clear that the curve started to decrease in the youngest group right after the intervention (blue line), then the decrease followed in the 12-19 (which were also directly affected by the intervention), and only subsequently in the adult population (grey line), likely indirectly benefitting from the decrease in contagion happening in the school-aged population.

Table 3 (previously Table S3 in Appendix, now moved to main text given its relevance), highlights how before the intervention contagion was spreading faster in the youngest age group compared to older groups, while after it there were no significant differences (see the relative hazards in the table). But again, the main finding is still related to the temporal ordering of events: contagion decreased first in the 3-11 and subsequently in other groups, an event that is hardly happened by chance.

5) Given the above, I think the authors’ conclusions are far too strong, and are not supported by the data they present. In particular, I think the following statement made by the authors is unsupported by their data: “Despite this, the claim that ‘schools are safe from COVID-19’, which was widely spread among the Italian media, appeared not to be supported by the empirical evidence.” (p. 16) (After reviewing their evidence, I am *more* inclined to agree that there was no impact of the school closures and that schools are safe). Additionally, the conclusion I noted in the summary, “it is nonetheless clear that school closure contributed substantially to mitigate the spread of the Alpha variant among the population” (p. 15), is also not supported by the authors’ data. I would suggest the authors remove these statements, and any other similar ones.

We hope our previous comments may have convinced the reviewers that the intervention played a role in reducing contagion. The contested quoting refers to the fact that some Italian media, also sometimes supported by members of the scientific community, used to claim before any intervention that schools were safe, not being a locus of contagion at all, and that school closure was not necessary. Our results go clearly against this unsubstantiated statement: as already highlighted above, we provided evidence that contagion was spreading more rapidly in school at the beginning of the month, as a consequence of a novel variant more contagious to kids, differently from previous strains. Independently of the effectiveness of the intervention, such evidence should be enough to show that COVID-19 was circulating in schools, which were not safe at all. How, in the middle of a COVID-19 wave, could a place where many people have to stay together in the same close and small environment for many hours, sometimes without masks (e.g. during meal hours), be safe from contagion? Schools were surely safe with previous COVID-19 strains, which affected children in a very marginal way, as reported by some studies and highlighted by local public opinion.
See for instance:
<https://www.corriere.it/politica/21_marzo_22/scuola-ricerca-dati-73-milioni-studenti-stare-classe-non-spinge-curva-pandemia-6d57776e-8a9c-11eb-82d5-215578033673.shtml>

<https://www.wired.it/attualita/scuola/2021/01/04/covid-19-iss-scuole/>

<https://www.orizzontescuola.it/wp-content/uploads/2021/01/Rapporto-ISS-COVID-19-n.-63_2020.pdf>

We absolutely do not claim that schools acted as a nidus of infection in all the pandemic phases, but it seems that in the presence of a novel variant spreading quickly also among kids, closing schools contributed to the containment of the local epidemic curve.

Minor comments

a) Would have been helpful if the draft had included page numbers. My page numbers are the page in the PDF file I was provided. We added them in the revised version.

b) The authors’ regression equation should have an exponential function in it given that it is supposed to be a poisson model, and should also have an error term. We fixed the equation.

c) P. 13: “Bearing in mind the average incubation period of the virus, we tested each model with a cut-off established at six days from the intervention date.” The authors could be clearer about what they mean by this. For a while as I was reading I thought it meant the authors lagged the data by 6 days.
This is typically done in the literature. As the virus has an incubation period, the effect of any intervention is likely to be observable after a minimum amount of time. If we assessed the effect of the intervention exactly from the day it was implemented we would have incurred a bias, as the new cases reported immediately thereafter were those of cases infected before the intervention. As reported in scientific literature cited in the text (see references 19-21), six days was the median incubation time, corresponding to the minimum amount of time necessary to observe the effect of the intervention.

d) Figure 2: I really don’t see a point to including the red-dashed lines. They just cause confusion. Plus I don’t think the authors ever really explain what it is.

Already in the text, page 9: “The dashed lines report the predictions from the adjusted models presented in Table 2, from which it could be difficult to grasp the change in the trend due to the adjustment for seasonality. Hence, the predictions from unadjusted models have been overlaid on the plot (continuous lines) to facilitate comprehension”.

Already in the figures captions: “Dashed line: unadjusted trend”.

e) Was school actually closed on March 5? It is not clear from the manuscript. That was a Friday, and though I am not sure if Italy follows the same system as the USA, but if schools were closed for Saturday and Sunday, maybe the authors should not consider the school closures to be implemented until March 8, the Monday in which might have been closed for the first time?
Yes, as reported in the text (page 3: “leading local authorities to impose a total school closure in Lombardy from March 5, which lasted until April 5”), March 5 corresponds to the closure day. Some schools are also open on Saturday in Italy, depending on the grade and the type of school. However, having introduced a six-days delay in the analysis of the intervention, this issue is already implicitly resolved, as we relied on March 11 as cut-off for the analysis, for the reasons described above.

f) P. 14: “Results are adjusted for seasonality and time-varying confounders, meaning that the effects detected are not attributable to some concurrent factors such as the underlying scenario applied, the epidemic trend in the whole country, or the number of swab tests performed locally or nationally.” – It is helpful to include these controls, but I think this statement is entirely too strong. One could argue that these controls are not sufficient to fully control for these factors (and could do so quite effectively, I might add), so I would not say these factors could not have *any* effect on the authors’ results. I would say that they include these controls which help address concerns that these factors could be the true causes of the authors’ estimates.

True. As discussed in another comment above, we agree that we are not able to control for every possible time-varying confounder, as in every statistical model it is not possible to include every relevant factor. We never claimed that we were able to completely isolate such effects in the model, we just mentioned those measures we were able to identify (thanks to the information available) and include in the models to adjust the estimates. There were already many passages in text (from Abstract to Discussion) in which we highlighted the fact that we were not able to “to completely discern the effect of school closures from concurrent policy measures”, and that “Being aware that the effects detected are not entirely ascribable to the intervention examined due to a portion of unadjustable confounders”, and similar.

We thank the referee for the comment, we amended the text, considering a more cautious statement (page 9): “Results are adjusted for some important concurrent factors, such as seasonality and time-varying confounders, meaning that the effects detected are not attributable to the underlying scenario applied, the epidemic trend in the whole country, or the number of swab tests performed locally or nationally. However, these controls cannot exclude the role of other unknown or unconsidered factors that could have influenced the trends (not available in data)”.

g) It is odd the way the authors at times reference the 12-19 age group yet don’t include their results in the paper (only an appendix). We agree that this group deserves more attention and in the restructuring of the manuscript we moved it in the main text, as suggested.
